# Iatrogenic foreign body in urinary bladder: Holmium laser vs. Ceramic, and the winner is…

**DOI:** 10.1590/S1677-5538.IBJU.2018.0229

**Published:** 2019-09-02

**Authors:** Daniele Castellani, Luca Gasparri, Redi Claudini, Maria Pia Pavia, Alessandro Branchi, Marco Dellabella

**Affiliations:** 1 Department of Urology, IRCCS-INRCA, Ancona, Italy

## Abstract

**Introduction:**

Urological surgery is estimated to be the third most common cause of iatrogenic-retained foreign bodies 1.

**Presentation:**

A 76-year old man was undergoing a transurethral resection of bladder tumor with a 26-Ch continuous flow resectoscope (Karl Storz, Germany). Before starting resection, a detachment of resectoscope sheath tip was noted. The ceramic tip was free-floating in the bladder lumen, and it would not fit within the sheath, making direct extraction using the loop impossible. An attempt was made to break it with a stone punch, but it was unsuccessful due to impossibility of closing it in the branches. Therefore, we decided to fragment the tip with holmium laser (RevoLix®, LISA Laser products, Germany), using an 800-micron, front-firing fiber. Laser device was settled at with 2.5 J energy and 5 Hz frequency. Ceramic appeared very hard, but it was difficult to carry on breaking with this setting because of tip retropulsion. Then, laser setting was switched to lower energy and higher frequency (1 J and 13 Hz). This setting guaranteed the same power of 13 W, but with minimal retropulsion.

**Results:**

Tip was fragmented against the posterior bladder wall in seven pieces, which were retrieved trough the outer sheath. A total 5.62 kJ were used to fragment it. At the end, superficial lesions of the posterior bladder wall were highlighted. Surgical time was 55 minutes. Patient was discharged home next day without problems.

**Conclusions:**

Holmium laser fragmentation is a safe and effective approach to remove foreign bodies from the bladder.

**ARTICLE INFO**

Available at: http://www.intbrazjurol.com.br/video-section/20180229_Castellani_et_al

Int Braz J Urol. 2019; 45 (Video #14): 853-853
